# Raw Cow Milk Protein Stability under Natural and Technological Conditions of Environment by Analysis of Variance

**DOI:** 10.3390/foods10092017

**Published:** 2021-08-27

**Authors:** Oto Hanuš, Josef Kučera, Eva Samková, Irena Němečková, Jindřich Čítek, Tomáš Kopec, Daniel Falta, Hana Nejeschlebová, Lucie Rysová, Marcela Klimešová, Ondřej Elich

**Affiliations:** 1 Dairy Research Institute Ltd., Ke Dvoru 12a, 160 00 Prague, Czech Republic; hanus.oto@seznam.cz (O.H.); nemeckova@milcom-as.cz (I.N.); hana.nejeschlebova@seznam.cz (H.N.); marcela.vyletelova@seznam.cz (M.K.); elich@milcom-as.cz (O.E.); 2 Czech-Moravia Breeders Corporation, Benešovská 123, 252 09 Hradištko, Czech Republic; kucera@cmsch.cz; 3 Department of Food Biotechnologies and Agricultural Products Quality, Faculty of Agriculture, University of South Bohemia in České Budějovice, Studentská 1668, 370 05 České Budějovice, Czech Republic; 4 Department of Genetics and Agricultural Biotechnology, Faculty of Agriculture, University of South Bohemia in České Budějovice, Studentská 1668, 370 05 České Budějovice, Czech Republic; citek@zf.jcu.cz; 5 Department of Animal Breeding, Faculty of Agronomy, Mendel University in Brno, Zemědělská 1665, 613 00 Brno-Sever, Czech Republic; tomas.kopec@mendelu.cz (T.K.); daniel.falta@mendelu.cz (D.F.); 6 Department of Food Science, Faculty of Agrobiology, Food and Natural Resources, Czech University of Life Sciences Prague, Kamýcká 129, 165 00 Prague 6, Czech Republic; rysoval@af.czu.cz

**Keywords:** cow, breed, farm factors, milk heat stability, milk composition, microbiologic indicators

## Abstract

Heat stability (HS) is substantial technology property of raw milk. Analysis of sources of HS variation and its regular monitoring can contribute to creating higher added value in the dairy industry. The goal of this analysis was to assess the practice sources of raw cow milk HS variability on the results of an extensive data set of bulk tank milk samples. There was implemented neither a compositional technology modification nor acidity adjustment of milk, just original raw milk was used for the analysis. A total 2634 HS analyses were performed, including other milk indicators, during three years of an experimental period. The log HS mean and standard deviation were 1.273654 ± 0.144189, equal to the HS geometric mean of 18.8 min. Explanation of the HS variability through the linear model used was 41.1% (*p* < 0.0001). According to the results of the variance analysis, the milk HS was influenced (*p* = 0.0033 and mostly <0.0001) by all the farm factors such as year; season; calendar month; altitude; total annual rainfall; herd size by the number of cows; milk yield; cow breed; type of milking; litter type in the stable; summer grazing application; farm effect. During the calendar months (*p* < 0.0001), milk HS values suggest similar seasonal dynamics with the somatic cell count, total count of mesophilic microorganisms, coli bacteria count and urea and lactose concentration and opposite configuration pattern to fat, crude protein, solids-not-fat and total solids content and milk freezing point depression. Here performed quantification of these effects by analyzing the variance may allow efficient raw milk selection to be processed into specific dairy products.

## 1. Introduction

The quality of raw milk is important for its technological processing on products [1]. Milk quality means, in particular, the sum of its hygiene and health indicators, such as the total count of microorganisms, the somatic cell count (SCC) or residues of inhibitory substances (RAD) [2]. In a broad sense, the quality of raw milk can also be described as its composition and several technological indicators. These may include titration acidity, fermentation ability, or cheeseability [3]. The values of mentioned indicators are then decisive for the processing of raw milk into dairy products. Dairy products with a long shelf-life must show good stability, mainly influenced by raw milk composition [4]. Thus milk quality is also important regarding the possibility of applying the added value in the dairying. In general, products with higher added value are increasingly required for economic reasons.

The ability of milk and concentrated milk to withstand a defined heat treatment without noticeable changes, such as flocculation of protein, is commonly denoted as heat stability [5]. Milk stability is considered the total time for visual coagulation to occur at a given pH and temperature, and it is directly related to the ability of milk to resist coagulation at certain temperatures [6]. As an indicator of protein stability, this heat stability (HS; also milk thermostability) is also an important technological property of raw milk as its evaluation can contribute to higher added value in dairy [7,8,9,10]. The composition of raw milk is essential for the stability of dairy products with a long shelf-life [4]. A sample of milk with short HS is generally considered to be unstable in terms of processability. It can result in problems during processing as opposed to longer HS [11,12]. Such milk has high stability. It means that it is ideal for the heat treatment processes to produce dairy products with a more extended shelf-life. The above mentioned is why it is crucial to study the sources of HS variability in practical conditions, although milk hygiene has improved during the last couple of decades, rendering milk less sensitive to coagulation [5]. Scientific and professional technological interest in the analysis of environmental (farm technology) and biochemical effects, including effects of food technology affecting the HS of lactoproteins also in the milk of small ruminants, can be found according to the other papers [13,14].

The HS is, therefore, substantial property of raw milk. This represents the resistance of lactoproteins against thermal coagulation [5]. In other words, it is the resistance of the milk to heat denaturation. Decreased milk quality ([15], for instance, because of mastitis) may adversely affect the HS. Hence, the raw milk’s good HS value is a prerequisite for milk processing into durable dairy products with long shelf life, such as condensed and sterilized, ultra-heat treated (UHT) milk [9,11]. Therefore, the HS was mentioned as an important indicator in evaluating milk quality, especially in terms of heat gains to which milk is exposed at its processing [16]. A simple technological test is usually used to determine the HS value. However, this test is laborious and often lengthy ([5] methods of subjective determination of HS). Because of HS determination laboriousness, the data sets used in the evaluation of HS variability are made up of a smaller number of data, which usually do not exceed one hundred [17,18]. Therefore, the number of HS measurements in our study is exceptional. Due to its laboriousness, HS measurements are often replaced by simpler and indirect determinations in practical dairy laboratories. This technological test is called determining the alcohol stability of milk. The results of the milk alcohol stability test may be in positive correlation with HS values (r = 0.28, *p* < 0.0001) [19], but this is not always the rule.

Furthermore, possible sources of variability were also analyzed in connection with this fact for the alcohol stability of raw milk [3,20,21,22,23,24,25,26]. Alcohol unstable milk showed higher SCC. On the contrary, the contents of lactose and casein were lower along with this [27]. According to these facts, high SCC could be marked as the primary causal reason, in this case of the high occurrence of alcohol unstable milk in commercial herds of dairy cows. Further, the differences in raw milk HS in dairy animals (among species such as cow, sheep, goat, camel, or yak) were also recorded [14,17,28,29]. Metabolic disorders of cows, such as subclinical rumen acidosis, could also reduce the technological quality of milk by reducing the content and quality of protein (so-called low protein syndrome; [30]).

In previous studies, we dealt with the relationships between milk indicators and the season to HS, and also the effect of feeding silage from legume-cereal mixtures on HS [31,32,33]. While the milk components of the bulk tank samples did not correlate much with the HS, in terms of simple correlations, closer relationships were shown between the composition of the milk and the HS, taking into account the effect of the season. Several HS studies have been performed during technological modifications of milk. While the change in calcium (CaCl_2_) level showed a high effect on the raw cow milk HS [4,34,35,36,37], the modifications of the citrate and urea concentration did not affect this HS value [4]. The HS of calcium citrate fortified milk was higher than the control milk and other calcium (different salts) fortified milk [38]. However, HS can be affected by many external and internal farm factors.

It turned out that in South America (Brazil, Chile, Colombia), the titratable acidity, alcohol stability, and HS of milk have been particularly intensively studied in recent years as the technological properties of milk. It was caused by often occurrence of problems of alcohol (heat) unstable raw milk that were not identified as acidic in cow herds. This phenomenon was probably determined by the problematic climate conditions for dairying, the progressive development of industrial milk processing there and the concomitant occurrence of possible technological problems. The effects of various dairy cow nutrition (starvation, feeding cows only by forage, anionic nutrition) on the stability of milk, concerning its titratable acidity, were evaluated [19,39,40]. In this context, a scheme [41] of a current analytical method for the identification of so-called UNAM bulk milk samples, i.e., unstable (positive ethanol test 72 °GI and negative boiling test) non-acidic cow milk samples (pH ≥ 6.6 or titratable acidity ≤ 18 °D) was described. In three feeding systems (herds) in spring-calving dairy cows, two based on grazing and one on a total mixed feed ration [42], no significant effects on the HS of milk in the mid-lactation (July) and in late lactation (September, October) were noted. Seasonal calving and pasture-based milk systems are widely used in countries with temperate climates and abundant rainfall [12]. As a result, synchronous changes in macro and micronutrients in milk are much more visible. Lactation here takes place in parallel with seasonal changes in the feed source, in contrast to non-seasonal milk production systems. It can have a greater impact on the processability and functionality of the milk. So, this logically means that the HS value of raw milk can be a general (global) problem in milk processing.

To the best of our knowledge, there is very little information on the variability of raw milk HS on farms in the scientific literature. Therefore, the goal of this work was to evaluate the practice sources which can influence the variability of raw cow milk HS. The priority was to carry out this evaluation with the results of an extensive data set of bulk tank milk samples, which were not under technological, compositional modification or acidity adjustment, just using original milk with its natural composition.

## 2. Materials and Methods

### 2.1. Dairy Cow Herds, Stables and Milk Sampling

Bulk tank milk samples were collected regularly on a monthly or two-week basis under dairy production conditions in East Bohemia. The following were included in the experimental assessment: 48 herds in total; 35 Czech Fleckvieh herds; 10 Holstein herds; 3 mixed herds. Cow milking was carried out by milking machine: 35 in the milking parlours (cows were housed in free cowsheds); 13 into the pipeline (cows were housed in binding stables). Dairy cows were milked twice a day. In monitoring, there were included 8928 dairy cows in total. In Table 1 there are listed relevant practice conditions in terms of environment and dairy technology in herds.

The experimental period took three years in total. During this time, 3310 bulk tank milk samples and 2829 for technological HS analyses were collected. Dairy cow feeding was performed regularly twice a day by TMR (total mixed ration). In this system of feeding, a mobile feed mixer tow trailer was used. The animals were fed using volume modification of the feed ration, according to the stage of lactation and milk yield. The composition of the feed rations was supplemented by the consumption of forage cereal concentrates, according to the standard feed tables for the current milk yield. The same method was used for the consumption of mineral feed supplements. The TMR quality during the experimental period can be considered medium, without extremes, regarding the roughage portion of feed rations. The grazing of cows on pasture was carried out during late spring, summer, and early autumn in a part of herds.

### 2.2. Quality Indicators for Bulk Tank Milk

Chemical preservation of bulk tank milk samples was performed with bronopol (2 bromine, 2 nitro, 1,3 propanediol, 0.03%, Broad Spectrum Microtabs). The samples were then transported to the laboratory under cold conditions (<8 °C). These were analyzed in an accredited dairy laboratory (Buštěhrad, Czech Republic), Czech-Moravia Breeders Corporation a.s. (ČMSCH) according to the relevant standard operation manuals. Milk indicators such as components and properties were determined by analytical methods and in units, according to the relevant abbreviations as listed in Table 2. Analyzes of milk samples were carried out using relevant methods and instruments that were calibrated and controlled by procedures and techniques according to standard operation manuals and with application of relevant result uncertainties of measurements stated by validation of methods in accredited laboratory.

The milk HS was logically analyzed in unpreserved samples. The HS results were methodically expressed in minutes as used units. The time was stopped at the moment of the creation of visible protein flakes in investigated milk [48], which means the visual denaturation. Milk samples were treated by heating in glass vials with thick walls in an oil bath at a temperature of 135 °C. This determination was performed with the volume of 2.5 mL of milk sample in the laboratory of the dairy plant Bohemilk Opočno. In this sense [49], a shortened test to determine milk resistance against protein denaturation by heating was also developed. Nevertheless, in this experiment, the complete method was used.

### 2.3. Statistic Assessment of the Results

Obtained experimental result file for milk indicators was added to records about conditions for raw milk production. These records were obtained by the specific questionnaire. Information about cow herds and relevant environmental and technological conditions were recorded and completed. A lack of information in the data file occurred, which was consequently limited to the complete form of results. Some monitored milk indicators have usually confirmed an absence of normal data frequency distribution, such as hygienic and microbiological indicators (SCC, total count of mesophilic microorganisms (TCMM), count of coliform bacteria (CCOL)) and also HS, according to the results of previous studies [31,32,33,50,51,52,53,54]. Further, because of this reason, the logarithmically transformed (log, on a decimal basis) results were used for the statistic evaluation, including an application of relevant geometric means. This procedure was logically carried out only with samples where a record of correspondence about type and time of analysis existed. So, by this method, the number (n) of bulk tank milk sample results in the evaluation was a little bit limited.

The statistic evaluation of experimental results was performed by multifactorial analysis of variance. There was used a reduced range of data file number (n = 2634 for HS compared to 3310 for other milk indicators). This reduction was done correctly in a mathematical sense with respect to reality. SAS ver. 9 program package [55] was used for the statistical result of processing of milk indicators. It resulted in Means and GLM (general linear model; the GLM procedure uses the method of least squares to fit general linear models) procedures. The significance of investigated factors was expressed on standard levels of probability. The results were processed by a linear model with fixed effects (Table 3). Including random effects, the model was performed with formula as follows (general equation parameters: Y = investigated milk indicator; µ = general mean; e_ijklmnopqrst_ = random effect):

$$Y_{\mathrm{ijklmnopqrst}}=\;\mu +{\mathrm{YR}}_{i}+{\mathrm{SE}}_{j}+{\mathrm{CM}}_{k}+{\mathrm{AL}}_{l}+{\mathrm{WE}}_{m}+{\mathrm{NC}}_{n}+{\mathrm{YI}}_{o}+{\mathrm{BR}}_{p}+{\mathrm{MT}}_{q}+{\mathrm{LT}}_{r}+{\mathrm{PA}}_{s}+{\mathrm{FM}}_{t}+e_{\mathrm{ijklmnopqrst}}$$

## 3. Results and Discussion

### 3.1. Main Statistic Results and Explanation Efficiency by Model of Analysis of Variance

Main statistic parameters of indicators in bulk tank samples of raw cow milk are included in Table 4. There is a good quality of raw milk included in cow herds during the experiment regarding dairy conditions compared to the country’s relevant references (The Czech Republic [56]). It is also well comparable to a high level of the west European results. Only two positive RAD cases were recorded (0.06%, n = 3310) in monitoring for three years of the experiment. It is approximately half of the value compared to the regular Czech Republic sliding average of 0.124 ± 0.033% (in the period from 2012 to 2016 [56]). That is why the RAD findings did not significantly affect the results of this evaluation negatively.

Explanation of the variability of the monitored milk indicators by the linear model (Table 4) ranged from 23.7 (for milk freezing point depression (MFPD)) to 59.5% (for solids-not-fat). The corresponding figure for the log HS was 41.1%. Thus, theoretically, 58.9% of the variability of HS values falls on uncontrolled effects. The efficiency of explanation of variability for all mentioned milk indicators, with the statistic model used, was significant (*p* < 0.0001). These figures are relatively high in terms of solving a biological problem and can be explained by including a specific farm effect that is the combined effect of all factors.

The data file used is interesting thanks to the high number of analytical results, duration of the experimental period, and the range of the evaluated farm conditions. The absolute majority of the observed fixed effects of the GLM significantly influenced most of the monitored milk indicators (Table 5). Milk HS was statistically significantly influenced by all the observed factors of farm conditions when the weakest effect (*p* = 0.0033) was in summer grazing or fresh green forage in the summer feeding ration.

### 3.2. The Effects of Practice Conditions on Milk Heat Stability

The log HS mean and standard deviation for this reduced analysis of variance of data file were 1.273654 ± 0.144189, which corresponds to a geometric mean (xg) of 18.8 min (Table 4). The year had a significant impact on HS values (Table 5, YR (year); *p* < 0.0001). The F criterion of analysis of variance was 75.78 (Table 6). Therefore, this effect was stronger concerning the F criterion, although the differences between years were practically relatively small.

The significant effect of the season on HS (Table 5, SE (season); *p* < 0.0001; F criterion 409.33, Table 6) proved to be the strongest factor. In the summer period, there are higher (better) HS values. It could be partly in accordance with other research opinions [57]. Seasonal variability in feed ration composition clearly affects HS, as seen in milk produced between November and March [58]. This period coincides with the indoor period of dairy cows. Milk from this part of the season has a shorter HS than milk from the rest of the year (April to October).

In contrast, better HS was observed in autumn and winter than in spring and summer following UHT ([59] in bulk raw cow milk). Nevertheless, in this context, the absence of an essential seasonal influence on most composition indicators, rennet gelation, and HS values suggest that milk from a mixed herd of cows with spring- and autumn-calving cows is suitable for cheese and milk powder production during a year [60]. However, following in-container sterilization, samples with added stabilizing salts showed significantly improved HS in autumn, whereas with added CaCl_2_, the best HS was observed in spring. The milk obtained in the autumn/winter season had significantly higher HS (*p* ≤ 0.01), with the most remarkable differences noted in the case of the Simmental cows [8].

The significant influence of altitude of dairy cow herd on HS (Table 5, AL (altitude); *p* < 0.0001; F criterion 29.51, Table 6) was moderate and showed the highest (best) HS values up to 300 m and the lowest at higher altitudes above 450 m.

The significant impact of the total annual rainfall on the HS (Table 5, WE (total annual rainfall); *p* < 0.0001; F criterion 9.7, Table 6) was less potent compared to the AL influence and showed the highest HS values for WE up to 450 mm, the mean at the highest WE over 650 mm and the lowest at medium WE. The finding of the highest HS values at the lowest WE corresponds to the same finding at lower altitudes, where the WE is usually lower compared to higher altitudes.

The significant influence of the herd size (Table 5, NC (number of dairy cows in the herds); *p* < 0.0001; F criterion 18.53, Table 6), which characterizes the production technology, was less pronounced on HS and indicated significantly lower HS in small herds up to 100 animals, mean HS in herds from 100 to 400 cows, and the highest HS in herds over 400 heads.

The significant effect of the herd milk yield on HS (Table 5, YI (level of milk yield by milk recording); *p* < 0.0001; F criterion 118.41, Table 6) was strong and demonstrated a significantly lower HS at a lower YI of up to 6000 kg of milk per lactation, mean HS at a YI of between 6000 and 9000 kg and a slightly higher HS at a high YI of over 9000 kg of milk per lactation in official milk recording procedure.

The significant impact of dairy cow breed on HS (Table 5, BR (breed); *p* < 0.0001; F criterion 12.86, Table 6) was less potent and confirmed slightly higher HS values for Czech Fleckvieh (CF) and mildly lower HS in Holstein (H) cows. Slightly lower was HS in other breeds (hybrids of CF and H and others). The lowest resistance to heat treatment was characteristic for milk of Polish Holstein-Friesian cows (average 120 s), the highest for Simmental (average 300 s), and the lactation phase did not affect HS of milk [10]. On the other hand [8], the best HS (*p* ≤ 0.01) was noted in the Black-and-White Polish Holstein-Friesian cows (220 s), while the milk of the Jersey cows was most susceptible to thermal destabilization (140 s).

The significant influence of the milking type on HS (Table 5, MT (type of milking); *p* < 0.0001; F criterion 15.23, Table 6) was relatively weak and evidenced the mean HS for automatic milking system (robot), higher when machine milking in the can and pipeline and lower at the milking parlour.

The significant effect of litter type in the stable on HS (Table 5, LT (litter type in the stable); *p* = 0.0001; F criterion 9.05, Table 6) was weak but showed a slightly lower HS in straw and mattress and slightly higher for technology separating liquid excrements.

The significant influence of the application of summer grazing and summer fresh green forage addition to total mixed ration in the course of dairy cow feeding on HS (Table 5, PA (application of summer grazing); *p* = 0.0033; F criterion 8.67, Table 6) was weak and indicated a lower HS for grazing and green feed. However, there might also exist a previous opposite standpoint in this evaluation. In the grazing system, the urea content of milk is usually increased during the spring and early summer season, which leads to its significantly higher HS compared to winter dry feeding [57]. Moreover, in Scotland, the recorded urea contents in milk accounted for most of the variability in HS during the year, where there were differences in the feeding of grazing cows in summer and autumn, which had higher HS than cows in a stable [61].

The significant impact of the farm (Table 5, FM (farm); *p* < 0.0001) on HS was expected due to specific conditions and their combinations at various localities. According to the F criterion 25.6 (Table 6) this FA effect was at medium power. Therefore, the selection of farms for the raw milk collection according to the history of higher HS values could be a method of effective dairy practice because of obtaining better raw material. This procedure is suitable for the improvement of operation certainty in the dairy plant during milk processing. On the other hand, the prediction of HS of concentrated (condensed) milk from the HS results of the corresponding unconcentrated (raw) milk for rapid quality testing purposes has been difficult, mainly due to different experimental conditions [5].

Concerning the future rapid practical monitoring of HS to select raw material from specific sources (farms), it turns out that [5] the infrared spectroscopy with Fourier transformation (MIR-FT) could be a successful procedure to elucidate the extent of changes in the secondary structure of crude protein during the heat treatment of milk and correlate them to the onset of coagulation and the quantity of aggregated protein.

### 3.3. The Seasonal Effect on Milk Indicators and Heat Stability

The mean values of milk indicators in Table 7 show seasonal trends. This effect of the CM (calendar month) was significant for all recorded milk indicators (Table 5) and especially for milk HS (*p* < 0.0001; Table 6, F criterion 8.25). It is clear that milk HS values suggest approximately similar seasonal dynamics with health and hygiene indicators, such as SCC, TCMM, and CCOL (Table 7; also [33]), which is not easily explainable and is it a little bit paradoxical but practically realistic. As expected, Machado et al. [19] reported negative relationships between HS and hygienic indicators TCMM and SCC (r = −0.15, *p* < 0.0003, n = 591 and r = −0.13, *p* = 0.0019, n = 591). However, this fact could be explained by the high mean values of TCMM and SCC in their file compared to our data set. Seasonal dynamics of hygienic indicators (TCMM and CCOL) were in accordance with the relevant type of research. As expected, there has been reported negative relationships [62]. Milk HS also had a similar trend with composition indicators such as urea and lactose content (Table 7; also [33]). However, other authors [63] did not find the urea effect on HS (*p* > 0.05) in individual milk samples. Another research found no correlation between urea and HS ([59] in bulk raw cow milk). For example, this does not correspond to the results reported by van Boekel et al. (1989, cit. [64]), where there is stated higher protein dissociation for higher urea content, and then casein molecules are more susceptible to flocculation. Other researchers [16] also did not find a significant impact of basic milk indicators on HS. Nevertheless, the approximately contradictory season trend of milk HS values was observed compared to fat, crude protein, solids-not-fat, total solids, fat/crude protein, fat/lactose, and MFPD (Table 7; also [33]). The component indicators seem to be more logical to link to HS dynamics [33]. Higher component concentrations can mark better visibility and traceability of the beginning of lactoprotein coagulation (denaturation, flakes) with extreme heat treatment of milk, which can demonstrate a simple technological relationship. This standpoint could also be supported with results reported by another research team [18], where artificial milk supplementation by milk proteins decreased milk HS. In general, some higher cow milk yield in the summer is also known and therefore, there is also an indication (Table 7) of a slightly positive relationship of milk HS to milk yield over calendar months.

### 3.4. Other Effects on Milk Heat Stability in Discussion

In many papers [18,22,65,66,67,68,69,70], HS was monitored in relation to technological modifications of milk, such as pH adjustment or other artificial additives, e.g., Ca (CaCl_2_), citrate, phosphates (different forms) with a targeted shift of milk buffering capacity (MBC) or whey protein and casein additions (technological improvement of raw material). The effects of elevated calcium, citrate, and urea levels on the stability of UHT milk stored for 52 weeks at 4, 20, 30, and 37 °C were investigated by Karlsson et al. [4]. An elevated level of calcium lowered the pH, resulting in sedimentation and significantly decreased stability. An elevated citrate level was associated with color, but the stability was not improved compared to the reference UHT milk. Elevated levels of urea or interaction terms had little effect on the stability of UHT milk.

Further, for example, protein additions (retentate) reduced milk HS [18]. However, it is not necessary to carry out this manipulation in all cases of milk processing. It means this is not always a positive trend to treat milk artificially before processing into food, especially regarding current sustainable views on healthy nutrition. Therefore, this thesis deals with the practical effects on HS of native milk in its natural composition and its original MBC.

**Figure 1:**
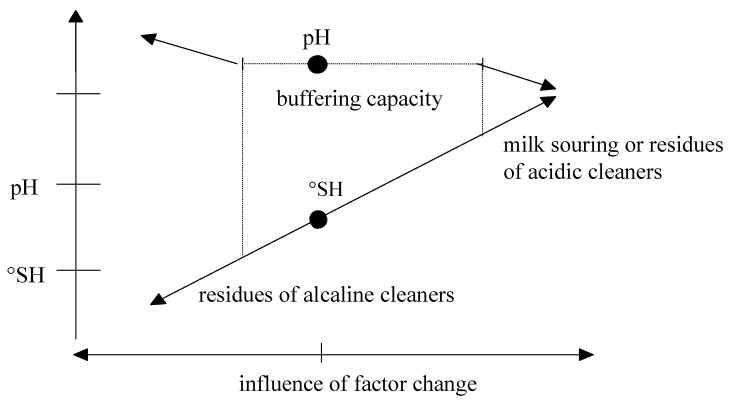
Scheme of milk buffering capacity (MBC) function. °SH = Soxhlet-Henkel degree of milk titration acidity; pH = milk active acidity.

Kailasapathy [71] referred to factors such as milk pH, salt content (can be adjusted by salt adding as stabilization [72]), urea, lactose, protein (and their variants), and also the season, lactation, and health of cows as essential for HS of milk proteins. As mentioned [11,71], pH acidity is the main factor in milk HS. HS variability in this evaluation of bulk milk samples was 39.6% (calculated from original values). In individual milk samples, it could be up to double by the qualified estimation. However, the pH variability for a large file of bulk milk samples was 1.9% (n = 2522, 0.13/6.82; [20]). That is 20.8 times less variability than HS, which is considerable. This low pH variability of raw milk is determined by its buffering capacity (Figure 1; MBC).

This MBC is more efficient towards the acidic area than the alkaline, as it is well known. It turns out that between values 1.9% (variability for pH acidity) and 39.6% (for HS), there is a large space for the explanation of sources to be filled since the quality of the raw material from the farm is a determining factor in the quality of dairy products. The multiple imbalances between HS and pH variability (39.6/1.9 = 20.8) when pH is the main factor of HS variability have to be better explained. It appears that this statement of pH, as the main HS factor, applies only to milk from a technological point of view, as such, it means in processing modifications and manipulations. However, according to the presented results in this work, there are several significant factors for the HS variability of raw milk in environmental and technological impacts on farms. Partial explanation and quantification of these impacts was the aim of this paper.

## 4. Conclusions

From the above experiment, it was concluded as follows:

- analysis of the variance on the influences of farm factors on the raw cow milk indicators, in particular the HS of lactoproteins, showed the severity of the influence of farm conditions on the quality of dairy products and the possibility of increasing the share of added value in the dairy industry;
- milk HS was statistically significantly influenced by all the farm factors (fixed effects of the linear model): year; season; calendar month; altitude; total annual rainfall; the number of dairy cows in the herd; milk yield level; cow breed; type of milking; litter type in the stable; summer grazing application; farm effect;
- quantification of these effects may allow efficient selection of raw milk during its collection in order to be processed into specific dairy products with the high-temperature treatment, according to the definition of farm conditions.

## Figures and Tables

**Table 1:** Basic indicators of herds of dairy cows included in the experiment.

Indicator	Unit	Minimum	Maximum	x ± s_d_
Mean number of cows per herd	head	4	630	186 ± 164
Altitude of herd	m	254	510	347.7 ± 68.8
Total annual rainfall, an average	mm	325	750	554 ± 143
Dairy plant raw milk delivery per herd, an average	kg	60	13,870	4454 ± 4095
Milk yield per cow and 305 days in milk, an average	kg	2033	11,124	6728 ± 2488

x ± s_d_ = arithmetic mean ± standard deviation; 305 days in milk = standard lactation.

**Table 2:** List of used milk analytical methods and indicators.

Milk Indicator	Abbr.	Unit	Method/Instrument	Added Information/Note
Fat	FA	%	CombiFoss FT+ (Foss, Hilleröd, Denmark)	MIR-FT
Crude protein	CRP	%	see above	total N × 6.38, MIR-FT
Lactose	LA	%	see above	monohydrate, MIR-FT
Solids-not-fat	SNF	%	see above	MIR-FT
Total solids	TOS	%	see above	MIR-FT
Urea	UR	mg·100 mL^−1^	see above	MIR-FT
Milk freezing point depression	MFPD	°C	see above	MIR-FT, combined with electrical conductivity measurement
Somatic cell count	SCC	10^3^·mL^−1^	see above	flow cytometry
Total count of mesophilic microorganisms	TCMM	10^3^ CFU·mL^−1^	IBC FC (Bentley Instruments, Chaska, MN, USA)	flow cytometry
Count of coli-form bacteria	CCOL	CFU·mL^−1^	plate cultivation method (VRBL agar, 37 ± 1 °C, abbreviated cultivation period 24–48 h)	
Residues of inhibitory substances	RAD	+/−	microbiological (*Geobacillus stearothermophilus*) inhibition assay (growth at 65 °C) with pH indicatorEclipse 50 (ZEU-INMUNOTEC, Zaragoza, Spain)	mostly as residues of antibiotic drugs and also for possible interference potential of some phytoactive substances
Fat/crude protein	FA/CRP		calculation	energy (ketosis) milk (cow) coefficient [43,44,45,46,47]
Fat/lactose	FA/LA		calculation	see FA/CRP

Abbr. = Abbreviation; MIR-FT = mid-infrared spectroscopy with Fourier-transformation; % = weight percentage (g·100 g^−1^); CFU = colony forming unit; +/− = positive/negative result.

**Table 3:** The list of used effects in statistic linear model equation of multifactorial analysis of variance and their specifications in the experiment.

Identification	Index	Effect Title	Number of Effect Categories (from–to)	Effect Specification
YR	i	year	1–3	
SE	j	season	1–2	1 = summer (from May to October); 2 = winter (from November to April
CM	k	calendar month	1–12	
AL	l	altitude	1–3	1 = <300; 2 = 300–450; 3 = >450 m
WE	m	total annual rainfall	1–3	1 = <450; 2 = 450–650; 3 = >650 mm
NC	n	number of dairy cows in the herd	1–3	1 = <100; 2 = 100–400; 3 = >400 of heads
YI	o	level of milk yield by milk recording	1–3	1 = <6000; 2 = 6000–9000; 3 = >9000 kg
BR	p	breed	1–3	1 = Czech Fleckvieh (CF); 2 = Holstein (H); 3 = various hybrids between CF and H
MT	q	type of milking	1–3	1 = machine milking into can and pipeline; 2 = milking parlour; 3 = automatic milking system
LT	r	litter type in the stable	1–3	1 = straw; 2 = rubber mattress; 3 = manure separation (liquid excrements)
PA	s	application of summer grazing (pasture)	1–2	1 = grazing, sometimes with green forage feeding next to silages in the mixture; 2 = without green forage, only by preserved roughage feeding rations (silages)
FM	t	farm	1–29	

Milk recording = 305 days in milk = standard lactation.

**Table 4:** Basic statistic parameters of raw milk indicators in bulk tank milk samples and efficiency explanation by model in analysis of variance.

IND	n	x	xg	s_d_	R^2^	*p*
FA	2993	3.89	-	0.282	0.463	<0.0001
CRP	3015	3.4	-	0.128	0.56	<0.0001
LA	3015	4.89	-	0.08	0.548	<0.0001
SNF	3015	8.89	-	0.144	0.595	<0.0001
TOS	2993	12.79	-	0.332	0.494	<0.0001
UR	1804	24.5	-	5.0	0.435	<0.0001
FA/CRP	2993	1.15	-	0.083	0.458	<0.0001
FA/LA	2993	0.8	-	0.063	0.477	<0.0001
MFPD	3015	−0.526048	-	0.005521	0.237	<0.0001
log SCC	3013	2.329102	213 ^a^	0.188909	0.344	<0.0001
log TCMM	3069	1.486766	30.6 ^b^	0.355881	0.302	<0.0001
log CCOL	3069	0.666409	4.6 ^c^	0.749125	0.244	<0.0001
log HS	2634	1.273654	18.8 ^d^	0.144189	0.411	<0.0001

IND = indicator; n = sample number; x = arithmetic mean; xg = geometric mean; s_d_ = standard deviation; R^2^ = coefficient of determination by linear model in analysis of variance; *p* = probability of zero hypothesis; ^a^ in 10^3^·mL^−1^; ^b^ in 10^3^ CFU·mL^−1^ (CFU colony forming unit); ^c^ in CFU·mL^−1^; ^d^ in minutes; used units and explanation of abbreviations of milk indicators are in Table 2; some figures in table were used also previously [31,32,33] in different evaluation of milk heat stability (HS).

**Table 5:** Fixed effects and their significance of impact, regarding monitored milk indicators by results of analysis of variance.

IND	YR	SE	CM	AL	WE	NC	YI	BR	MT	LT	PA	FM
FA	<0.0001	<0.0001	<0.0001	<0.0001	<0.0001	<0.0001	<0.0001	<0.0001	0.0184	*0.2236*	<0.0001	<0.0001
CRP	<0.0001	<0.0001	<0.0001	<0.0001	0.0001	<0.0001	<0.0001	<0.0001	0.0023	*0.8489*	*0.8016*	<0.0001
LA	<0.0001	<0.0001	<0.0001	<0.0001	<0.0001	<0.0001	<0.0001	<0.0001	<0.0001	*0.1398*	*0.5349*	<0.0001
SNF	<0.0001	<0.0001	<0.0001	<0.0001	*0.2389*	<0.0001	<0.0001	<0.0001	*0.3563*	*0.8043*	*0.2625*	<0.0001
TOS	<0.0001	<0.0001	<0.0001	<0.0001	<0.0001	<0.0001	<0.0001	<0.0001	0.0112	*0.2698*	<0.0001	<0.0001
UR	<0.0001	<0.0001	<0.0001	<0.0001	<0.0001	<0.0001	<0.0001	<0.0001	0.0003	*0.0948*	<0.0001	<0.0001
FA/CRP	<0.0001	<0.0001	<0.0001	<0.0001	<0.0001	<0.0001	<0.0001	0.0004	<0.0001	*0.2148*	<0.0001	<0.0001
FA/LA	<0.0001	<0.0001	<0.0001	<0.0001	<0.0001	<0.0001	<0.0001	<0.0001	*0.6359*	*0.4766*	<0.0001	<0.0001
MFPD	<0.0001	*0.2197*	0.0187	<0.0001	<0.0001	<0.0001	<0.0001	0.0001	<0.0001	<0.0001	<0.0001	<0.0001
log SCC	<0.0001	<0.0001	0.0012	<0.0001	<0.0001	<0.0001	<0.0001	0.0154	<0.0001	*0.3618*	<0.0001	<0.0001
log TCMM	<0.0001	<0.0001	0.0328	<0.0001	<0.0001	<0.0001	<0.0001	<0.0001	<0.0001	*0.8379*	*0.429*	<0.0001
log CCOL	<0.0001	<0.0001	<0.0001	<0.0001	<0.0001	<0.0001	<0.0001	*0.7686*	<0.0001	*0.2288*	<0.0001	<0.0001
log HS	<0.0001	<0.0001	<0.0001	<0.0001	<0.0001	<0.0001	<0.0001	<0.0001	<0.0001	0.0001	0.0033	<0.0001

IND = indicator; figure means probability of zero hypothesis by F value; normal letters, statistic significant; italics letters, insignificant; the explanation of abbreviations of milk indicators (x axis of this table) and environmental and technology farm factors (y axis) is in Table 2 and Table 3; some figures in table were used also previously [31,32,33] in different evaluation of milk heat stability (HS).

**Table 6:** Influences on raw cow milk heat stability, according to various environmental and farm technology factors by results of analysis of variance.

FAFA	FAFAT	F Criterion	IFA	*t* Value/Probability
YR	i	75.78	1	−6.83/<0.0001
			2–3	−1.15/0.2503
SE	j	409.33	1–2	5.87/<0.0001
AL	l	29.51	1	8.42/<0.0001
			2–3	5.95/<0.0001
WE	m	9.7	1	7.28/<0.0001
			2–3	−4.76/<0.0001
NC	n	18.53	1	−9.49/<0.0001
			2–3	−3.81/0.0001
YI	o	118.41	1	7.39/<0.0001
			2–3	2.33/0.0201
BR	p	12.86	1	1.19/0.2334
			2–3	0.27/0.7836
MT	q	15.23	1	1.11/0.2669
			2–3	−3.72/0.0002
LT	r	9.05	1	−0.93/0.3536
			2–3	−0.43/0.6679
PA	s	8.67	1–2	−5.76/<0.0001
FM	t	25.6	1–29	-

F = criterion value as an influence power; FAFA = farm factor; FAFAT = farm factor type; IFA = identification of farm factor type; *t* = *t* criterion value; the explanation of abbreviations of environmental and technology farm factors is in Table 3.

**Table 7:** The trend dynamics of means of milk indicators along calendar months by results of analysis of variance.

IND	1	2	3	4	5	6	7	8	9	10	11	12
FA ^x^	3.87	3.82	3.8	3.69	3.76	3.65	3.64	3.63	3.74	3.89	3.81	3.89
CRP ^x^	3.39	3.34	3.34	3.29	3.3	3.24	3.22	3.2	3.28	3.4	3.38	3.4
LA ^x^	4.87	4.89	4.86	4.88	4.92	4.95	4.94	4.92	4.92	4.89	4.88	4.89
SNF ^x^	8.87	8.83	8.81	8.79	8.82	8.79	8.75	8.7	8.79	8.89	8.91	8.89
TOS ^x^	12.74	12.65	12.6	12.48	12.58	12.45	12.39	12.34	12.53	12.79	12.72	12.79
UR ^x^	24.76	24.31	25.63	26.29	26.55	25.55	27.49	26.07	26.85	24.49	24.33	24.49
FA/CRP ^x^	1.14	1.15	1.14	1.13	1.14	1.13	1.13	1.14	1.14	1.15	1.12	1.15
FA/LA ^x^	0.8	0.78	0.78	0.76	0.76	0.74	0.74	0.74	0.76	0.8	0.78	0.8
MFPD ^x^	−0.52625	−0.52707	−0.52662	−0.52691	−0.52722	−0.5277	−0.52665	−0.52694	−0.52672	−0.52605	−0.52727	−0.52605
SCC ^xg^	219	212	214	208	212	230	226	251	229	213	218	213
TCMM ^xg^	27.2	24.6	25.3	27.6	29.3	34.7	33.1	31.4	33.2	30.7	27.6	30.7
CCOL ^xg^	5.8	3.7	4.5	4.8	6.2	9.1	10.1	8.0	8.2	4.6	3.7	4.6
HS ^xg^	16.2	16.8	18.6	19.8	18.9	18.8	19.4	20.2	20.9	18.8	19.0	18.8

IND = indicator; ^x^ = arithmetic mean; ^xg^ = geometric mean; 1–12 = calendar months; used units and explanation of abbreviations of milk indicators are in Table 2.

## Data Availability

The data presented in this study are available on request from the corresponding author.
